# Low-Level Laser Therapy Activates NF-kB via Generation of Reactive Oxygen Species in Mouse Embryonic Fibroblasts

**DOI:** 10.1371/journal.pone.0022453

**Published:** 2011-07-21

**Authors:** Aaron C-H. Chen, Praveen R. Arany, Ying-Ying Huang, Elizabeth M. Tomkinson, Sulbha K. Sharma, Gitika B. Kharkwal, Taimur Saleem, David Mooney, Fiona E. Yull, Timothy S. Blackwell, Michael R. Hamblin

**Affiliations:** 1 Wellman Center for Photomedicine, Massachusetts General Hospital, Boston, Massachusetts, United States of America; 2 Programs in Leder Human Biology and Translational Medicine, and Biological Sciences in Dental Medicine, Harvard University, Cambridge, Massachusetts, United States of America; 3 Program in Oral and Maxillofacial Pathology, Harvard School of Dental Medicine and Brigham and Women's Hospital, Boston, Massachusetts, United States of America; 4 Harvard School of Engineering and Applied Sciences, Cambridge, Massachusetts, United States of America; 5 Department of Dermatology, Harvard Medical School, Boston, Massachusetts, United States of America; 6 Aesthetic and Plastic Center, Guangxi Medical University, Nanning, People's Republic of China; 7 Smith College, Northampton, Massachusetts, United States of America; 8 Aga Khan Medical College, Karachi, Pakistan; 9 Department of Medicine and Cancer Biology, Vanderbilt University School of Medicine, Nashville, Tennessee, United States of America; 10 Harvard-MIT Division of Health Sciences and Technology, Cambridge, Massachusetts, United States of America; Penn State Hershey Cancer Institute, United States of America

## Abstract

**Background:**

Despite over forty years of investigation on low-level light therapy (LLLT), the fundamental mechanisms underlying photobiomodulation at a cellular level remain unclear.

**Methodology/Principal Findings:**

In this study, we isolated murine embryonic fibroblasts (MEF) from transgenic NF-kB luciferase reporter mice and studied their response to 810 nm laser radiation. Significant activation of NF-kB was observed at fluences higher than 0.003 J/cm^2^ and was confirmed by Western blot analysis. NF-kB was activated earlier (1 hour) by LLLT compared to conventional lipopolysaccharide treatment. We also observed that LLLT induced intracellular reactive oxygen species (ROS) production similar to mitochondrial inhibitors, such as antimycin A, rotenone and paraquat. Furthermore, we observed similar NF-kB activation with these mitochondrial inhibitors. These results, together with inhibition of laser induced NF-kB activation by antioxidants, suggests that ROS play an important role in the laser induced NF-kB signaling pathways. However, LLLT, unlike mitochondrial inhibitors, induced increased cellular ATP levels, which indicates that LLLT also upregulates mitochondrial respiration.

**Conclusion:**

We conclude that LLLT not only enhances mitochondrial respiration, but also activates the redox-sensitive NFkB signaling via generation of ROS. Expression of anti-apoptosis and pro-survival genes responsive to NFkB could explain many clinical effects of LLLT.

## Introduction

Low level light (or laser) therapy (LLLT) has been used for more than forty years to promote healing, reduce pain and inflammation, and prevent tissue death [1], [2]. Despite many basic and clinical reports, the therapy remains controversial largely due to uncertainties about the fundamental molecular and cellular mechanisms responsible for transducing signals from the photons incident on the cells to the biological effects that take place in the irradiated tissues.

It has been reasonably well established that mitochondria are a principal intracellular target of red and near-infra-red light [3]. Cytochrome C oxidase (unit IV of the mitochondrial respiratory chain) is a chromophore that absorbs light as far into the infra-red as 1000 nm [4]. There have been reports of increased cytochrome c oxidase activity after LLLT [5] and many reports of increased ATP synthesis after light delivery to isolated mitochondria [6]. Additional evidence of the role of cytochrome c oxidase as a chromophore in LLLT has been provided by action spectra studies from Karu's laboratory in Russia [7] and from Eells and Wong-Riley in Wisconsin [8]. Many genes have their transcription upregulated (or down regulated) after illumination of cells with various wavelengths and fluences of light. For instance, illumination of human fibroblasts with 628 nm light emitting diode led to altered expression of 111 genes (68 up, 43 down) that can be sub-categorized into 10 functional groups [9].

Nuclear factor kappa B (NF-kB) is a transcription factor regulating expression of multiple genes [10], and has been shown to govern various cellular functions, including inflammatory and stress-induced responses and survival [11]. NF-kB activation is regulated by negative feedback mediated by IkB, an inhibitor protein that binds to NF-kB, but can undergo ubiquitination and proteasomal degradation [12], thus freeing NF-kB to translocate to the nucleus and initiate transcription [13]. NF-kB is a redox-sensitive transcription factor [14], that has been proposed to be the sensor for oxidative stress [15]. Reactive oxygen species (ROS) can both activate NF-kB directly [16], and ROS are also involved in NF-kB activation by other stimuli such as tumor necrosis factor alpha (TNFα), phorbol ester, and interleukin (IL)-1 [17]. Several laboratories have observed the formation of ROS in cells in vitro after LLLT [18], [19], [20], [21], and it has been proposed that ROS are involved in the signaling pathways initiated after photons are absorbed by the mitochondria within cells [22].

In the present report, we describe the effect of light from an 810 nm laser on mouse embryonic fibroblasts (MEF) isolated from a transgenic NF-kB luciferase reporter (HLL) mouse [23]. These mice have been genetically engineered so that luciferase expression is driven by the NF-kB-dependent portion of the human immunodeficiency virus-1 long terminal repeat. They have been used to carry out molecular imaging using a bioluminescence camera of inflammation after various stimuli such as tumor necrosis TNFα, lipopolysaccharide (LPS) and IL-1 [24]. We reasoned that these cells would be ideal to test the hypothesis that LLLT activates NF-kB to mediate various downstream biological processes.

## Results

### Activation of NF-kB by laser *in vitro*

**Figure 1:**
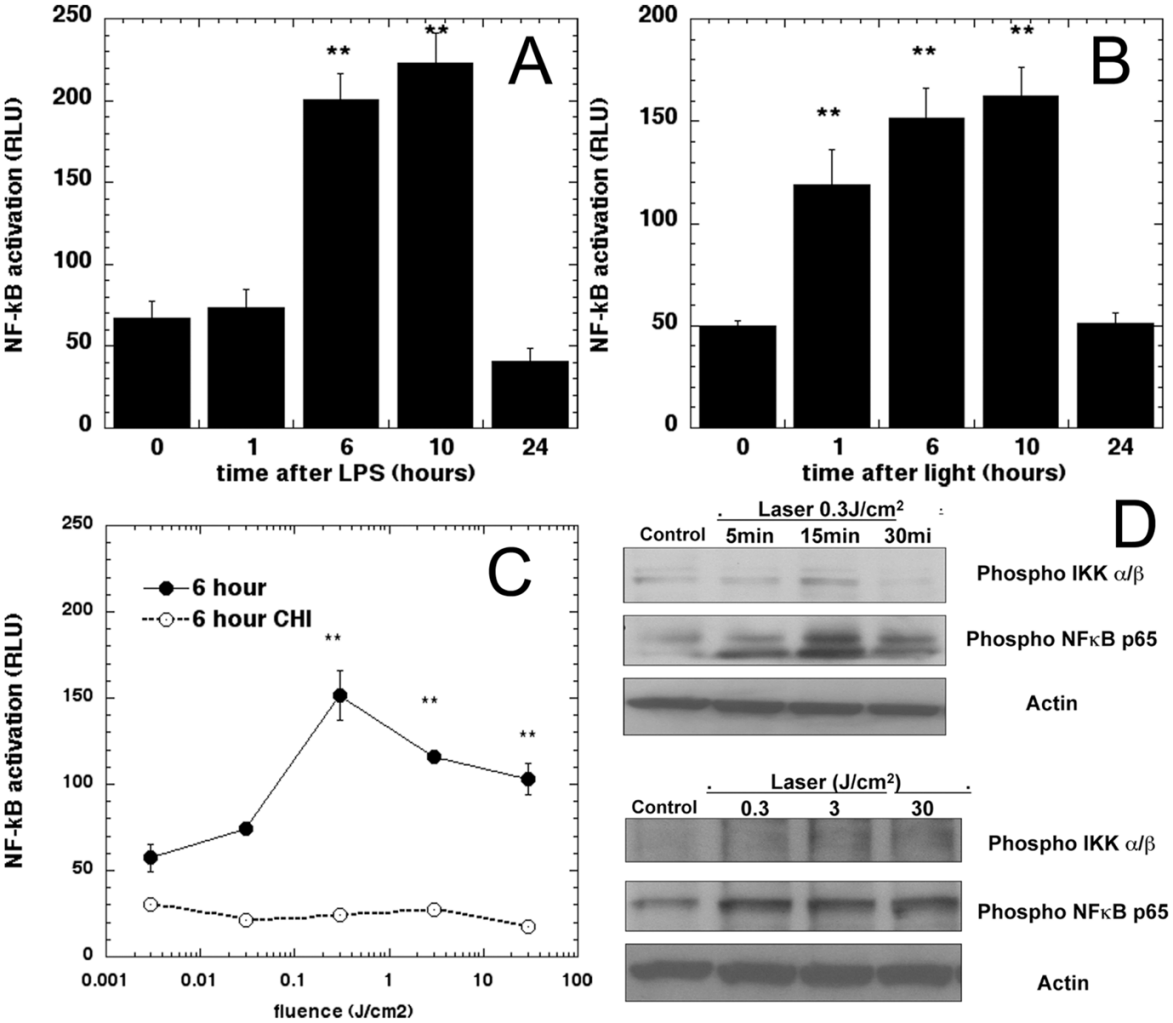
Laser activates NF-kB in MEF. (A) Time course of NF-kB activation measured by luminescence after addition of 0.5 ug/mL of LPS (B) Time course of NF-kB activation after 0.3 J/cm2 810-nm laser irradiation. (C) NF-kB measured 6 hours post irradiation with a wide range of 810-laser fluences and effect of protein synthesis inhibitor cycloheximide. * p<0.05; ** p<0.01 compared to baseline. (D) Immunoblotting for Phospho NFκB and IκB α/β following laser irradiation of MEFs at 0.3 J/cm^2^ over time (upper panel) and at various fluences 0.3, 3 and 30 J/cm^2^ at 15 min (lower panel) was performed. Normalization for protein loading with Actin is shown.

We used an agonist of toll-like receptor 4 signaling, the bacterial product lipopolysaccharide to confirm activation of NF-kB activation in the isolated primary MEFs. The results (Fig. 1A) show an increased NFkB expression at 6 hours following LPS addition that increased further till 10 hours before returning to baseline at 24 hours. We then irradiated cells with 0.3 J/cm^2^ of 810 nm laser and measured luciferase response at the same time points (Fig. 1B). We observed a distinct NF-kB luciferase increase as early as 1 hour post-laser and the responses at 6, 10 and 24 hours were similar to those seen with LPS. The light fluence response of NF-kB measured at 6 hours is shown in Fig. 1C (solid line). We observed the strongest signal at 6 hours with 0.3 J/cm^2^ but the luminescence decreased somewhat (but was still significantly above baseline) when the energy density was higher. To confirm that the luminescence increase measured was due to the increased expression of luciferase enzyme activated by NF-kB binding to the HIV long terminal repeat alone and not a direct effect of laser irradiation on the transgene, we pre-incubated Luc MEF with the protein synthesis inhibitor cycloheximide (10 µM for 2 hours) and then laser irradiated with same power densities and measured the luminescence at 6 hours. We observed that after cycloheximide, luminescence at all fluences was depleted to a steady level, about 30% of baseline level (Fig. 1C broken line).

NF-kB activation proceeds via phosphorylation of its inhibitor, IKK α/β which leads to its ubiqitination and degradation, thus allowing phospharylation of the p65 subunit which then translocates to the nucleus to induce specific gene expression. To confirm that these biochemical pathways are activated by LLLT, we performed western blot analysis of MEF cell extracts at 5, 15 and 30 minutes after treatment with 0.3 J/cm^2^ of 810-nm laser, and with different fluences (0.3, 3, and 30 J/cm^2^) at 15 minutes (Fig. 1D). We observed the distinct phosphorylation bands of both IKK α/β and NF-kB p65 as early as 5 minutes post light and were maintained for 30 minutes further validating the luciferase reporter results.

### Laser increases ROS production

**Figure 2:**
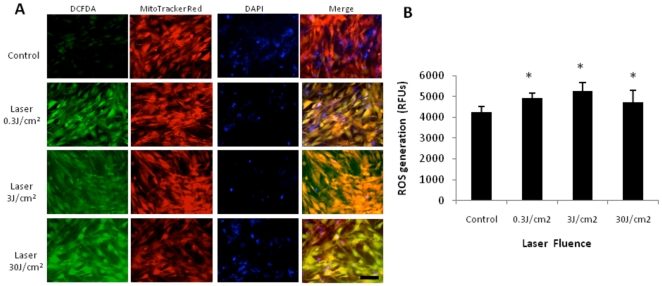
Laser increases ROS in MEF. (A) MEF were treated with 810 nm laser at varying fluencies 0, 0.3, 3 and 30 J/cm^2^ and ROS generation was measured with CM-H_2_DCFDA (green). Mitochondrial localization is demonstrated with MitoTracker Red (red) and overlay (yellow) while cell nuclei were strained with DAPI (blue) (B) Amplex Ultrared assay demonstrates quantitative increase in ROS following laser irradiation at varying fluences. * p<0.05.

Many groups have reported the increase of cellular reactive oxygen species (ROS) after LLLT in vitro [19], [25], [26], [27], [28], [29]. Since one of the important initiators of NF-kB activation in several cell types is known to be via ROS generation [15], we checked to if ROS were increased after laser irradiation at varying fluencies in our MEF NF-κB Luc cells using fluorescent reporters namely, CM-H_2_DCFDA, MitoTracker and DAPI (Fig. 2A). While 0.03 J/cm^2^ gave little fluorescence above background (data not shown), 0.3, 3 and 30 J/cm2 gave a robust increase in green fluorescence. Since the primary source of ROS within cells is superoxide generated in the mitochondria [30], we used the MitoTracker Red to demonstrate co-localization of the laser generated ROS with CM-H_2_DCFDA as evidenced by the overlay (Fig. 2A). Quantitative validation of this result was performed with the Amplex Ultrared assay that demonstrated increased ROS generation at the three fluences used (Fig. 2B).

### Laser increases ATP synthesis

**Figure 3:**
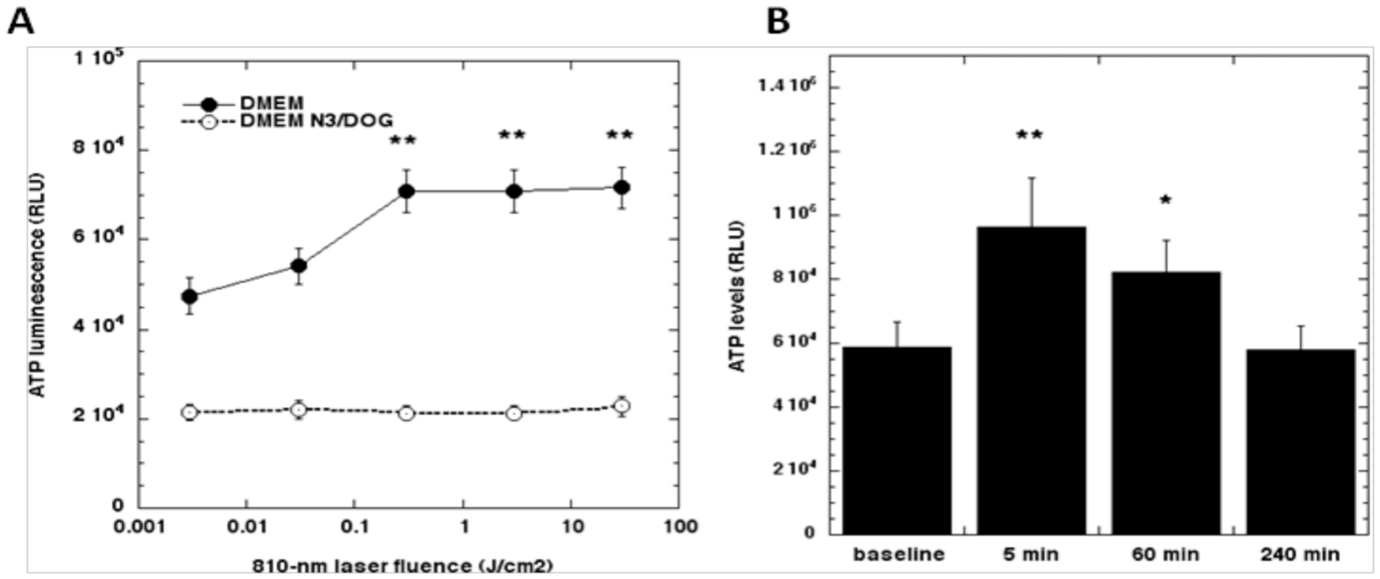
Laser increases ATP synthesis in MEF. (A) ATP increase measured at 5 min post LLLT with wide range laser fluences and effect of mitochondrial inhibitors (sodium azide/deoxyglucose). (B) Time course of ATP increase after 0.3 J/cm2 810 nm laser. * p<0.05; ** p<0.01 compared to baseline.

To test if the NF-Κb Luc MEF respond to 810 nm laser irradiation with increased ATP, we first irradiated the cells with fluences varying over 5 orders of magnitude (0.003 to 30 J/cm^2^) and measured ATP levels over time. The results measured at 5 minutes post-light showed no increase with 0.003 J/cm^2^, a small increase at 0.03 J/cm^2^ and a large increase that formed a plateau with fluences of 0.3, 3 and 30 J/cm^2^ (Figure 3A solid line). The time course of ATP increase reached a peak immediately after a 5-minute irradiation and gradually declined to baseline levels in 6 hours (Figure 3B). To confirm specificity, we pre-incubated the cells for 2 hours with sodium azide and deoxyglucose, widely used as a mitochondrial inhibitor and an inducer of chemical hypoxia [31], in the media and then performed laser irradiation and observed ATP generation. The metabolic inhibitors not only lowered the baseline levels of ATP but also made the cells unresponsive to the laser induced ATP generation (Fig. 3A dashed line).

### Mitochondrial inhibitors increase ROS and NF-kB activation but not ATP

**Figure 4:**
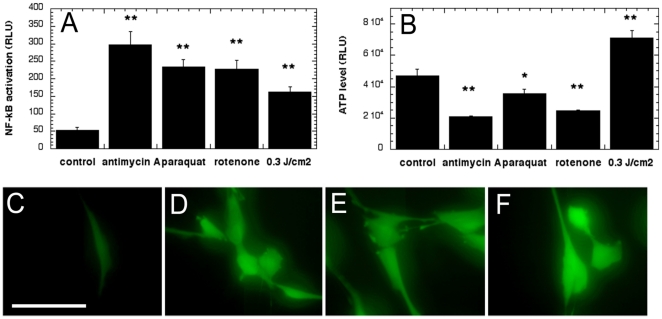
Mitochondrial inhibitors increase ROS and NF-kB activation in MEF but reduce ATP. (A) NF-kB activation measured at 6 hours after 2 hour incubation with 100 µM inhibitors, (B) ATP levels measured after 2 hours incubation with 100- µM inhibitor. * p<0.05; ** p<0.01 compared to control. Inhibitors (100 µM) were added for 2 hours and CM-H_2_DCFDA was used to measure ROS generation at 30 min (C) control, (D) antimycin A, (E) rotenone, (F) paraquat.

We tested three mitochondrial inhibitors that have all been reported to induce intracellular ROS by blocking the normal flow of electrons in the mitochondrial respiratory chain[32], [33], [34]. We reasoned that if the mechanism of LLLT activation of NF-kB was via generation of ROS, then these mitochondrial inhibitors should be able to activate NF-kB as well. We observed that all the inhibitors after 2 hour incubation showed NF-kB activation at the 6-hour time point (Figure 4A). Paraquat and rotenone showed similar NF-kB activation perhaps due to the fact that they are both complex I inhibitors, and antimycin A, a complex III inhibitor, induced the strongest activation. We also measured ATP changes in the cells with these inhibitors and found that, in contrast to 810 nm laser, they depleted ATP as might be expected from their ability to inhibit mitochondrial respiration by cutting electron transport at various sites (Figure 4B). Figure 4C-F confirms that these inhibitors increased ROS levels in MEF cells as determined by increased fluorescence intensity of CM-H_2_DCFDA at 30min following treatment.

### Antioxidants abrogate NF-kB activation but not ATP after laser

**Figure 5:**
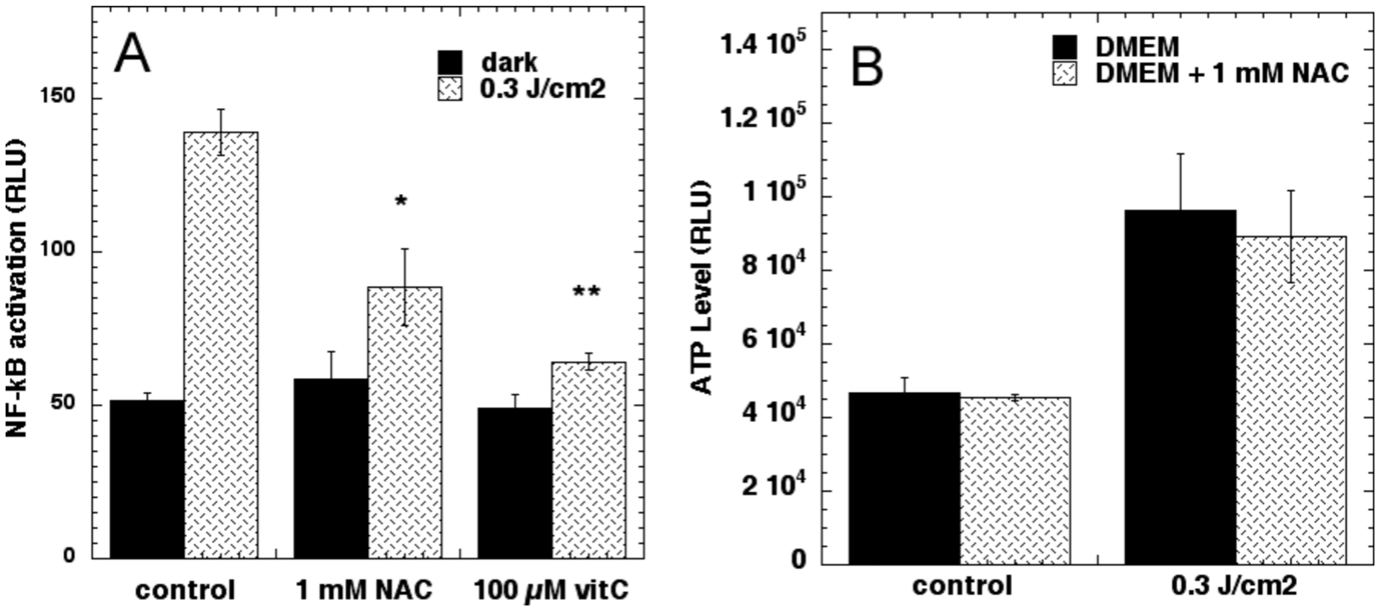
Antioxidants abrogate laser-induced NF-kB activation in MEF but not ATP increase. (A) NF-kB measured 6 hours post irradiation of cells incubated 2 hours with 1 mM N-acetyl cysteine or 100 µM ascorbic acid. (B) ATP measured after 2-hour incubation with 1 mM N-acetyl cysteine. * p<0.05; ** p<0.01 compared to control.

To further confirm that the activation of NF-kB is due to laser-generated mitochondrial ROS we pre-incubated the antioxidants, N-acetyl-L-cysteine (NAC) and ascorbic acid and performed LLLT and luciferase assays. The results shown in Fig. 5A showed that both ascorbate and to a lesser extent NAC significantly reduced the laser induced increase in NF-kB activation but had no significant effects on the control. We then asked whether the antioxidants had any effect on the laser-induced increase in ATP. As seen in Fig. 5B neither pre-incubation with NAC (nor ascorbate - data not shown) did not have any effect on the increased ATP generation following laser irradiation at 0.3 J/cm^2^.

## Discussion

This report has demonstrated for the first time that low levels of near-infrared laser light can activate NF-kB in primary MEF cells. We confirmed reports from other laboratories [19], [25], [26], [27], [28], [29] that visible (and NIR light) can induce expression of ROS in illuminated cells. Our results indicate that activation of NF-kB after 810 nm laser light is mediated via ROS generation. The fact that the addition of antioxidants abrogates the activation of NF-kB provides additional evidence that ROS are involved in the activation of NF-kB. There are both similarities and differences between the cellular effects of NIR laser and inhibitors that produce mitochondrial ROS by inhibiting complexes of the respiratory chain. Both laser and mitochondrial inhibitors produce ROS and activate NF-kB, but laser increases ATP while the inhibitors decrease ATP. The fact that antioxidants do not abrogate the ATP increase suggests that the action of light increases electron transport, which in the absence of antioxidants can cause increased electron leakage producing superoxide.

The very wide range of fluences (3 orders of magnitude between 0.03 and 30 J/cm^2^) that gave positive NF-kB activation suggests that the phenomenon is akin to a switch being turned on after a minimum light dose in the region of 0.03 J/cm^2^ has been delivered as has been observed with other LLLT induced biological processes such as latent TGF-β1 activation [35]. The time course of the laser-induced NF-kB activation we observed appeared to be significantly different from that observed with conventional LPS activation [36]. Following laser irradiation, we observed significant activation at 1 hour while LPS did not show any significant increase until 6 hours. It has been recently discovered that there are (at least) two NF-kB activation pathways [37]. The canonical NF-kB signaling pathway activated in response to infections (toll like receptor signaling) and cytokines is based on degradation of IkB inhibitors. This pathway depends on the IkB kinase (IKK), which contains two catalytic subunits, IKKα and IKKβ. IKKβ is essential for inducible IkB phosphorylation and degradation, whereas IKKα is not. IKKα is involved in processing of the NF-kB2 (p100) precursor. IKKα preferentially phosphorylates NF-kB2, and this activity requires its phosphorylation by upstream kinases, one of which may be NF-kB-inducing kinase (NIK). IKKα is therefore a pivotal component of a second NF-kB activation pathway based on regulated NF-kB2 processing rather than IkB degradation.

Recent work has identified protein kinase D (PKD, formerly known as PKCν) as a mitochondrial sensor of oxidative stress [38]. It was reported that low dose hydrogen peroxide (250-nM) led to tyrosine phosphorylation at Tyr463 on PKD and the enzyme's consequent activation [39]. This activation of PKD then led to activation of IKKβ, followed by IkB degradation and NF-kB activation [40]. The pathway did not depend on IKKα nor on NF-kB inducing kinase (NIK) [39].

Considering the fact that NF-kB is known to be a redox-sensitive nuclear transcription factor [15] it is reasonable to conclude that laser induced NF-kB is due to the ability of the laser to produce mitochondrial ROS. The primary ROS generated in the mitochondrial electron transport chain is superoxide, which is destroyed by superoxide dismutase (SOD). SOD converts superoxide to a freely diffusible molecule, hydrogen peroxide that is capable of oxidizing membrane lipids to lipid peroxides that then oxidize the dihydrofluorescein probe [41].

Karu *et al.* proposed a novel mitochondrial signaling pathway in mammalian cells initiated by red and near-IR light in vitro in 2004 [42]. One hypothesis to explain the effect of NIR light on cells is the absorption of the photons by cytochrome c oxidase (CCO), which is unit IV of the respiratory chain [6], [42], [43]. The action spectrum of light on several cellular functions has been observed to closely resemble the absorption spectrum of CCO [7], [8]. One theory that has yet to be conclusively be demonstrated is that nitric oxide can be photodissociated from CCO [44]. NO is known have a function as a regulator of respiration [45]. Hu *et al.* [46] showed that He-Ne laser illumination increased mitochondrial membrane potential together with ATP synthesis in melanoma cells. They also observed upregulation of cytochrome c oxidase activity, increased phosphorylation of Jun N-terminal kinase (JNK) that later activated activator protein-1 (AP-1) which, in turn, led to increased cell proliferation. He-Ne laser irradiation in isolated mitochondria has been shown to promote O_2_ consumption by cytochrome c oxidase and increase electron transport [5].

NF-kB is a highly pleiotropic transcription factor [47] that induces expression of many gene products associated with known beneficial effects of LLLT. For instance anti-apoptotic and pro-survival proteins, proteins involved in cellular proliferation and migration and increased collagen synthesis and myofibroblast differentiation respond to NF-kB activation [48].

One interesting point that arises from our results is that NF-kB activation is known to be pro-inflammatory [49] while numerous reports indicate that LLLT has a pronounced anti-inflammatory effect. Recently Mafra de Lima et al reported [50] that the beneficial effects of LLLT in reducing inflammation in rat bronchial segments could be abrogated by a NF-kB inhibitor. A report by Moriyama et al [51] showed that LLLT increased inducible nitric oxide synthase expression in a mouse model of arthritis consistent with NF-kB activation. Another possibility is that the initial response to cell stress typical of NF-kB activation induces a productive response that includes lower NF-kB activation when measured at a later time point when the light has had a therapeutic effect. It is also possible that the initial pro-inflammatory response after LLLT induces expression of eicosanoids such as resolvins designed to end inflammation [52].

A limitation of our study is that we only showed laser-induced NF-kB activation in fibroblasts. It is possible that other cell types particularly those involved in inflammatory responses may show a different pattern of NF-kB activation after LLLT.

## Materials and Methods

### Ethics statement

All animal procedures were approved by the Subcommittee on Research Animal Care (IACUC) of Massachusetts General Hospital under protocol number 2009N000006 and met the guidelines of National Institutes of Health.

### Reagents

2-Deoxy-L-glucose, sodium azide, cycloheximide, antimycin A, rotenone, N-acetylcysteine, ascorbic acid, lipopolysaccharide from *Escherichia coli* and penicillin-streptomycin were from Sigma-Aldrich (St Louis, MO). Fluorescent probes 5-(and 6) chloromethyl-2′,7′-dichlorodihydrofluorescein diacetate acetyl ester (CM-H_2_DCFDA) and MitoTracker Red were from Invitrogen Molecular Probes (Invitrogen, Carlsbad, CA). Steady-Glo Luciferase Assay and Cell Titer Glo ATP Assay were from Promega (Madison, WI). Bradford protein assay (BCA) was from Pierce Biotechnology Inc (Thermo-Scientific, Rockford, IL).

### Cell isolation and culture

Timed pregnant female HLL (NF-kB luciferase reporter) mice [23] were used in the study. The animals were housed with a 12-hour light/dark cycle with access to food and water *ad libitum*. Mouse embryonic fibroblasts (Luc MEF) were isolated from embryos removed from pregnant mice between day 13 and day 15 according to the protocol described by Sun and Taneja [53]. The cells were then cultured in Dulbecco's-modified Eagle's medium (DMEM) (Gibco, Invitrogen, Carlsbad, CA) supplemented with 10% fetal bovine serum (Hyclone, Waltham, MA) and 1% penicillin-streptomycin (Gibco, Invitrogen, Carlsbad, CA), in 37°C incubator. For all the experiments, cells were grown to about 80% confluence before seeding to 96 well plates and only the cells between passage 3 and passage 8 were employed.

### Laser irradiation

The experiments were conducted with a diode laser (Model D030-MM-FCTS/B, Opto Power Corp, Tucson, AZ), which emits 810 nm near infrared radiation. Power densities generated in a round homogeneous spot of diameter 3-cm were measured with a power meter (Model DMM 199 with 201 Standard head, Coherent, Santa Clara, CA) and ranged from 1 mW/cm^2^ to 30 mW/cm^2^. Light was delivered for times varying from 7 seconds to 5 minutes to deliver different energy densities, namely 0.003, 0.03, 0.3, 3 and 30 J/cm^2^. Wells were irradiated in blocks of 3 X 3cms by use of a mask covering the plate.

### Assessing ROS generation

Cells were plated in chamber slides (Nunc, Thermo-scientific, Rockford, IL) and incubated in 37°C incubator overnight, washed three times with PBS, and then changed to a PBS solution (with calcium and magnesium) (Cellgro, Manassas, VA) with 10 µM CM-H_2_DCFDA, 500 nM MitoTracker Red and 300 nM DAPI (Molecular Probes, Invtirogen, Carlsbad, CA) for 15 min and mounted on the microscope stage. In one experiment CM-H_2_DCFDA was added at different times after illumination and incubated for a further 30 min in every case to determine kinetics of ROS generation following laser irradiation (data not shown). Laser irradiation at various fluencies were performed on the samples and immediately imaged for fluorescence using an inverted fluorescence microscope (Olympus IX81, Centerway, PA) with a spinning disc laser system (BD Carv II, BD Biosciences, Franklin Lakes, NJ) with equal exposure times and images were collected with IP lab (Ver 4.0, Exton, PA).

For Quantitative ROS measurements, the Amplex UltraRed (Moelcular Probes, Invitrogen, Carlsbad, CA) was used. Briefly, the cells were seeded in a 96 well black walled plate with clear bottoms (Costar, Corning, NY) and laser irradiated at varying doses followed by addition of PBS (with calcium and magnesium) (Cellgro, Manassas, VA) with 100 µM Amplex Ultrared and 0.2 U/ml Horse radish peroxidase (both Molecular Probes, Invtirogen, Carlsbad, CA) for 30 min. Fluorescence was measured using a microplate reader (Synergy HT, Bio-Tek, Winooski, VT).

### Luciferase Assay for NF-kB activation

MEFs were plated in a 96 well plates (Costar, Corning, NY) with 3000 to 5000 cells per well and were treated with either LLLT or 0.5 µg/mL LPS, washed with PBS and at lysed with 50 µl passive lysis buffer (Promega, Madison, WI) at various time points post treatment. Lysates were transferred to 96 well white plates and substrate was added (Promega, Madison, WI) to assess luciferase activity with a microplate reader (Synergy HT, Bio-Tek, Winooski, VT). The remaining lysate was used in a Bradford assay (BCA Assay, Pierce Biotechnology Inc, Thermo-Scientific, Rockford, IL) to estimate total protein content for normalization. Cycloheximide (10 µM) (Calbiochem, EMD Biochemicals, Gibbstown, NJ) was added 2 hours before treatments to inhibit protein synthesis.

### Immunoblotting

Following laser irradiation, cells were washed with cold PBS and lysed (175 mM NaCl, 25 mM HEPES pH 7.4, 10% Glycerol, 5 mM EDTA, 1% Triton X, 50 mM Sodium Fluoride, 5 mM Sodium Orthovandate) with Complete Mini Protease inhibitor (Roche). The cells were then scarped using a plastic cell lifter, transferred to labeled microfuge tubes and spun at 14000 rpm at 4°C for 15 min. The clear supernatant was transferred to a fresh tube and total protein was estimated by Bradford assay (BCA Assay, Pierce Biotechnology Inc, Thermo-Scientific, Rockford, IL) and electrophoresis was performed with precast Tris-Glycine gels (Invitrogen, Carlsbad, CA), transferred onto 0.2 µm nitrocellulose membranes (Invitrogen, Carlsbad, CA), blocked with 3% bovine serum albumin (Sigma-Aldrich, St Louis, MO) and incubated with primary antibodies Phospho-NFkB (Ser 536), Phospho IkB α/β (both Cell Signaling, Beverly, MA) and Actin (Chemicon) overnight at 4C followed by appropriate species-specific secondary antibody (Jackson ImmunoResearch Laboratories Inc, West Groove, PA). Following rigorous washes with Tris Buffered Saline and Tween, chemiluminescence substrate (Pierce Biotechnology Inc, Thermo-Scientific, Rockford, IL) was added to the membrane and detected by films (Kodak MR, Sigma-Aldrich, St Louis, MO).

### Cell-Titer Glo Assay for ATP

MEFs were plated in a 96 well plates (Costar, Corning, NY) with 3000 to 5000 cells per well. At the various time points following LLLT, 50 µL cell lysis buffer was added and plates were placed on shaker for 2 minutes to ensure completely release of ATP. 95 µl lysates were then transferred to luminescence 96 well plates (White plate, Costar, Corning, NY) and 100 µL of Cell-Titer Glo Assay mix was added into each well, shaken for 5 minutes to stabilize the luminescence signal and measured with a plate luminometer (Wallac Tri-Lux beta, PerkinElmer Life and Analytical Sciences, Waltham, MA). 5 µl of the lysate was used to estimate total protein (BCA Assay, Pierce Biotechnology Inc, Thermo-Scientific, Rockford, IL). Another set of cells was incubated with 5 mM 2-deoxy-L-glucose and 0.05% sodium azide for two hours before laser irradiation to inhibit ATP synthesis. In some experiments, antioxidants N-acetylcysteine (1 mM) and ascorbic acid (100 µM) were added for 2 hours before laser irradiation.

### Statistical Analysis

All assays were performed in multiple wells (n = 9) and Microsoft Excel (Redmond, WA) software was used for Single-Factor ANOVA to evaluate the statistical significance of experimental results (p<0.05).
